# The development and validation of the Addiction-like Eating Behaviour Scale

**DOI:** 10.1038/ijo.2017.158

**Published:** 2017-08-08

**Authors:** H K Ruddock, P Christiansen, J C G Halford, C A Hardman

**Affiliations:** 1 Department of Psychological Sciences, University of Liverpool, Liverpool, UK; 2 UK Centre for Tobacco and Alcohol Studies, UK

## Abstract

**Background:**

Overeating and obesity are frequently attributed to an addiction to food. However, there is currently a lack of evidence to support the idea that certain foods contain any specific addictive substance. An alternative approach is to focus on dimensions of observable behaviour, which may underpin a behavioural addiction to eating. To facilitate this, it is necessary to develop a tool to quantify addiction-like eating behaviour, which is not based on the clinical criteria for substance dependence. The current study provides initial validation of the Addiction-like Eating Behaviour Scale (AEBS).

**Methods:**

English speaking male and female participants (*N*=511) from a community sample completed the AEBS, alongside a range of other health- and eating-related questionnaires including the Yale Food Addiction Scale (YFAS) and Binge Eating Scale (BES). Participants also provided their height and weight to enable calculation of body mass index (BMI). Finally, to assess test–retest reliability, an additional 70 participants completed the AEBS twice, 2 weeks apart.

**Results:**

Principle components analysis revealed that a two-factor structure best accounted for the data. Factor 1 consisted of items that referred to appetitive drive, whereas factor two consisted of items that referred to dietary control practices. Both subscales demonstrated good internal reliability and test–retest reliability, and a confirmatory factor analysis confirmed the two-factor scale structure. AEBS scores correlated positively with body mass index (BMI) (*P*<0.001) and other self-report measures of overeating. Importantly, the AEBS significantly predicted variance in BMI above that accounted for by both the YFAS and BES (*P*=0.027).

**Conclusions:**

The AEBS provides a valid and reliable tool to quantify the behavioural features of a potential ‘eating addiction’. In doing so, the AEBS overcomes many limitations associated with applying substance-dependence criteria to eating.

## Introduction

Worldwide rates of obesity have more than tripled in the past three decades.^1^ This recent rise in obesity is often attributed to the ‘addictive’ qualities of certain foods, and a popular theory holds that some people may develop an ‘addiction’ to food and eating.^2^ However, although reward mechanisms common to addiction are, to an extent, also associated with control of eating behaviour, the validity of the ‘food addiction’ concept, and the way in which it should be defined and assessed, continues to be widely debated.^3, 4, 5^

Previous definitions and assessments of food addiction, such as the Yale Food Addiction Scale (YFAS), rely upon the Diagnostic Statistical Manual (DSM)-IV-TR and DSM-5 criteria for substance-dependence/substance-use disorder.^6, 7^ However, the applicability of these criteria to the assessment of eating behaviours is limited by several fundamental differences between drugs and food. Most notably, there are neurobiological differences between the effects of drugs and food (for example, refs 8, 9), and drug use is thought to have more potent effects on the neurological processes involved in motivated behaviour relative to palatable food consumption.^10^ Furthermore, several of the symptoms listed in the DSM-IV and 5 criteria for substance-dependence/substance-use disorder appear less applicable to the assessment of problematic eating. For example, addiction-like eating may not entail ‘impairment to daily functioning’ or the cessation of ‘important social, occupational or recreational activities*’*. Notably, however, the less stringent diagnostic criterion set out in the DSM-5, which requires the presence of two out of 11 symptoms, would more easily permit a diagnosis of food addiction in the absence of these particular symptoms (relative to the DSM-IV which requires three out of seven symptoms to be present). For a full discussion regarding the physical and societal differences between drugs and food, the reader is referred to review articles by Hebebrand *et al.*^4^ and Ziauddeen *et al.*^5^

The limited comparability between drugs and food places constraints upon the ecological validity of the YFAS, which is largely dependent on a substance-based model of food addiction.^11^ As such, several authors have suggested the need to develop a more precise operational definition of food addiction that is not reliant upon existing conceptualisations of substance-based addictions.^3, 4, 5^ To develop a novel framework for ‘food addiction’, one approach is to focus on dimensions of observable behaviours, which may underpin a behavioural addiction to eating.^4^ Indeed, the view that ‘food addiction’ may be best conceptualised as a behavioural, rather than substance-based, ‘eating addiction’ represents the consensus opinion of a number of researchers in this area (for example, ref. 12). This approach circumvents the assumption that certain foods contain specific ‘addictive’ substances, and has implications for the potential inclusion of ‘addictive eating’ within future editions of the DSM, which now provides a category for non-substance-based addictions. Although gambling is the only behavioural addiction currently recognised within this category, there is scope for the inclusion of other maladaptive behaviours. It is therefore necessary to identify exactly which behaviours and cognitions may underlie maladaptive addiction-like patterns of eating, and to develop a method of assessing their severity.

Dual-process theories of motivation propose that appetitive reward systems interact with regulatory systems to control behaviour.^13^ Specifically, there is extensive evidence indicating that an increased responsivity to reward-related cues, coupled with a diminished ability to exert ‘top–down’ inhibitory control over these responses, is an underlying risk factor for the development of addictive behaviours.^13, 14, 15^ For example, Tarter *et al.*^15^ found that the presence of inhibitory control deficits during childhood significantly predicted the onset of substance-use disorders in young adulthood. Consistent with this and in relation to eating, a prospective study reported greater weight gain, over a 1-year period, in those with an increased preference for snack foods and a lower capacity for inhibitory control, compared with those with higher inhibitory control.^16^ It has also been shown that food reward responsivity positively predicts body mass index (BMI), but only when impulsiveness is also high, providing further support for the dual-system model in relation to overweight and obesity.^17^ Taken together, these findings are consistent with the notion that overeating and addictive behaviours, such as drug use, are characterised by core behavioural processes (‘addiction-like eating behaviour’).^10^ An important distinction however is that, unlike drug use, eating is essential for survival and, as such, heightened reward responsivity to food may often be an adaptive mechanism (for example, following chronic food restriction). We conceptualise ‘addiction-like eating’ as referring specifically to maladaptive eating behaviours, which place individuals at higher risk of overweight and obesity.

Drawing on the above, the aim of the current research was to develop a questionnaire to quantify addiction-like eating behaviours. To facilitate this, in a previous qualitative study, we used an inductive approach to identify behaviours that are commonly associated with ‘food addiction’ amongst young adults residing in the UK.^18^ Participants (*N*=210) were asked to indicate whether or not they perceived themselves to be ‘food addicts’, and to provide a brief explanation for their response. Thematic analysis revealed six characteristics that were commonly associated with food addiction in both self-perceived food addicts and non-addicts. These included: (a) a tendency to eat for reward rather than physiological need, (b) persistent food cravings, (c) an inability to control oneself around food, (d) a preoccupation with food and eating, (e) increased weight or an unhealthy diet and (f) a particular problem controlling one’s intake of foods high in fat, salt and/or sugar. Using these qualitative data, and guided by the previous theoretical approaches and empirical findings described above, the current study developed and provided preliminary validation of the Addiction-like Eating Behaviour Scale (AEBS).

## Materials and methods

### Participants

Participants (*N*=511) were recruited via public advertisements that were displayed on various social media websites (for example, Facebook and Twitter) and on the internal web pages of the University of Liverpool, UK. The sample size was based upon recommendations that there should be between 5 and 10 observations for each item included in a factor analysis.^19^ In exchange for taking part, participants were given the chance to enter a prize draw to win £50, and/or were allocated course credits. All participants who were over the age of 18 and fluent in English were eligible to take part. Given that addiction-like eating may be particularly prevalent in those with pathological eating patterns,^20, 21^ we decided not to exclude those with a history of eating disorders. This is consistent with the approach used to validate the YFAS.^6^

Prior to analysis, data pertaining to individual participants were randomly allocated into one of two groups from the main data set (group 1 or group 2). Initial exploratory factor analysis and internal reliability analyses were performed using responses from group 1 (*n*=307). Responses from group 2 (*n*=204) were used to confirm the factor structure. Further analyses of the scale’s convergent, divergent and incremental validity were performed using combined responses from both groups. Finally, a separate sample of 70 participants (group 3) was recruited to assess the test–retest reliability of the AEBS. Ethical approval was obtained from the University of Liverpool Research Ethics Committee and all participants provided informed consent prior to taking part in the study.

### Measures

#### Addiction-like eating behaviour scale

The original pool of 62-items that were assessed for inclusion in the AEBS were derived from qualitative responses obtained from a previous study.^18^ To ensure that items adequately captured a range of addiction-like eating behaviours, we included at least five items to capture each ‘theme’ that was identified in the previous study. Specifically, items referred to either: (1) A tendency to eat for reward rather than physiological need (for example, ‘I continue to eat despite feeling full’), (2) Persistent food cravings (for example, ‘I crave certain foods’), (3) An inability to control oneself around food (for example, ‘I find it difficult to limit what/how much I eat’), (4) A preoccupation with food and eating (for example, ‘I spend lots of time planning my meals’), (5) Increased weight or an unhealthy diet (for example, ‘I am unable to control my weight’) and (6) A particular problem controlling ones intake of foods high in fat, salt and/or sugar (for example, ‘I have a particular problem controlling myself around foods that are high in fat, sugar and/or salt’). For each item, participants indicated the extent to which they agreed with the statement, or the frequency by which they engaged in the given behaviour. Responses were provided using 5-point Likert scales which ranged from ‘Strongly Disagree’ to ‘Strongly Agree’ or from ‘Never’ to ‘Always’.

#### Assessments of convergent and divergent validity

The following scales were included to assess the convergent validity of the AEBS, and were therefore expected to correlate positively with the scale: (1) Yale Food Addiction Scale (YFAS^6^); (2) Binge Eating Scale (BES^22^); (3) Emotional Eating Scale (EES^23^); (4) Eating Troubles Module (EAT-26^(ref. 24)^). We also included an assessment of self-perceived food addiction, which has previously been found to significantly predict the rewarding value of food and *ad-libitum* calorie intake.^25^ Please see online Supplementary Materials for more information about these measures.

To assess the scale’s divergent validity, the following assessment tools were included: (1) Rutgers Alcohol Problem Index (RAPI^26^), (2) Behavioural Inhibition System/Behavioural Approach System Reactivity (BIS/BAS^27^). These scales were not expected to correlate with AEBS scores. See online Supplementary Materials for more information about these measures.

All of the above scales, with the exception of the assessment of self-perceived food addiction, were included in the previous validation of the YFAS^6^ and so we opted to include them here for consistency.

### Procedure

Groups 1 and 2 completed the questionnaires online at www.qualtrics.com. After providing informed consent, questionnaires were completed in the following order: AEBS, the assessment of self-perceived ‘food addiction’, BES, EAT-26, YFAS, EES, RAPI and BIS/BAS. Participants then provided demographic information including their age, gender, weight (in kilograms, pounds or stones) and height (in centimetres, or feet and inches). Finally, participants who wished to be entered into the prize draw provided their e-mail address. To obtain test–retest data, participants in group 3 completed paper-based versions of the AEBS twice, 2 weeks apart. As in groups 1 and 2, participants in group 3 were also asked to provide their age, gender, weight and height, and were fully debriefed following the study. In all three groups, height and weight data were self-reported.

### Data analysis

Data were analysed using SPSS Statistics version 22 (Armonk, New York, USA) and AMOS version 22 (AMOS, Chicago, IL, USA).

#### Pre-analysis checks and data preparation

Prior to analysis, participants’ responses on each of the AEBS items were assigned a value of 1 to 5 (1=strongly disagree/never, 2=disagree/rarely, 3=neither agree or disagree/sometimes, 4=agree/most of the time, 5=strongly agree/always). As higher scores indicated greater addiction-like eating tendencies, some items were reverse scored so that inter-correlations with other items remained positive. AEBS items were assessed for skewness and kurtosis, and sampling adequacy was checked using the Kaiser–Meyer–Olkin (KMO) statistic. Bartlett’s test of sphericity was used to assess whether correlations between items were sufficiently large for principle components analysis (PCA) (values *P*<0.05 are indicative of sufficient inter-item correlations).

#### Exploratory factor analysis (group 1)

A parallel analysis (using the Monte-Carlo simulation method^28^), and a scree-plot^29^ were used to identify an initial factor solution. A PCA with an oblique rotation (as factors were expected to correlate with each other^30^) was then conducted, and items were removed if they had factor loadings of <0.40,^31^ or had loadings of more than 0.35 on more than one factor.^32^ Items that had low item-total correlation (<0.40^33^) or did not share a conceptual meaning with the remaining items in a scale^34^ were also removed following reliability analysis (Cronbach’s alpha).

#### Internal consistency and descriptives (groups 1 and 2)

Cronbach’s alpha was used assess the internal consistency of each AEBS subscale with *α*=0.70 considered an acceptable lower bound.^35^ AEBS total and subscale scores were computed by summing values (that is, 1–5) that corresponded to participants’ responses to each item. Independent *t*-tests assessed whether AEBS total or subscale scores differed between males or females, and Pearson’s correlations were used to examine whether scores were associated with age and BMI. All analyses were conducted for groups 1 and 2 separately.

#### Confirmatory factor analysis (group 2)

Using AMOS 22,^36^ a Confirmatory Factor Analysis was performed on the solution with best fit. Items were free to load onto their corresponding latent factors, and latent factors were free to correlate with each other. Model fit was assessed by examining the normed *χ*^*2*^ statistic (*χ*^2^/df),^37^ Goodness of Fit Index (GFI^38^), Comparative Fit Index,^39^ the Root Mean Square Error of Approximation (RMSEA^40^ and Standardized Root Mean Square Residual (SRMR^41^). Normed *χ*^2^/df ratios of <2,^37^ and GFI and CFI values of above 0.90,^38, 39^ are deemed acceptable. RMSEA values indicate either good fit (<0.05), fair fit (>0.05, <0.08), mediocre fit (>0.08, <0.10) or poor fit (>0.10),^40^ and SRMR values of <0.08 are considered good fit.^41^ Where appropriate, model fit was improved by adding covariance pathways between error terms. These were determined following inspection of the modification indices.

#### Convergent and divergent validity (groups 1 and 2)

Correlational analyses were conducted to assess the convergent validity of the AEBS compared with other eating behaviour scales (that is, YFAS, EES, BES, EAT-26) and BMI. A logistic regression was used to determine the extent to which AEBS scores could predict whether or not respondents perceived themselves to be food addicts. To examine the scale’s overlap with the YFAS, a linear regression was conducted to examine the extent to which the presence (or absence) of each YFAS symptom-predicted scores on each subscale of the AEBS. Results from this analysis are provided in the online supplementary analysis. Divergent validity was assessed by comparing correlations between the AEBS total score and problematic alcohol use (assessed using the RAPI), and behavioural inhibition/activation (BIS/BAS). Please see online Supplementary Materials for further discussion regarding these findings.

#### Incremental validity (groups 1 and 2)

A hierarchical linear regression was conducted to assess whether the AEBS could account for additional variance in BMI beyond that predicted by the YFAS symptom count and BES. A hierarchical logistic regression was also conducted to explore whether the AEBS could predict self-perceived food addiction over and above YFAS symptom count and BES scores. In both models, YFAS symptom count and BES scores were included in step 1, whereas total AEBS scores were entered into step 2. Finally, an ordinal regression was conducted to evaluate the scale’s ability to predict weight classification. Participants were grouped as either underweight (BMI ⩽18.49 kg m^−2^), normal weight (18.50–24.99 kg m^−2^), overweight (25.00–29.99 kg m^−2^) or obese (BMI ⩾30 kg m^−2^). Weight classification was entered as the dependent variable (with ‘underweight’ as the reference category), and BES, YFAS symptom count and AEBS scores were entered as covariates.

#### Test–retest reliability (group 3)

Using data from group 3, test–retest reliability was assessed by examining the intra-class correlation between AEBS total and subscale scores obtained at the initial time of testing and following the 2-week interval. Scores of 0.60 or more indicate good test–retest reliability.^42^

## Results

### Pre-analysis checks and participant characteristics

Values of skewness and kurtosis ranged between the acceptable levels of −2 and 2, thus no transformations were necessary.^43^ The KMO statistic for the model was above the acceptable level of 0.05 (KMO=0.93) and Bartlett’s test of sphericity was significant (*P<*0.001). Participant characteristics for each of the two groups are shown in Table 1.

### Exploratory factor analysis (group 1)

The parallel analysis and scree-plot initially identified a five-factor solution. However, subsequent PCA with oblique (oblimin) rotation revealed no clear 5-factor solution. Following removal of items (using the procedure outlined in the data analysis section), a two-factor solution was derived from the remaining 15 items, with eigenvalues 6.64 and 1.96 for factors one and two, respectively. Factor one comprises 9 items that referred to appetitive drive (for example, I continue to eat despite feeling full), and accounted for 44.26% of the total variance. Factor 2 comprises 6 items that referred to low dietary control (for example, Despite trying to eat healthily, I end up eating ‘naughty’ foods) and accounted for 13.04%, of the total variance. Factors 1 and 2 were moderately positively correlated with each other (*r*=0.523, *P<*0.001). Item-factor loadings are provided in Table 2. The full 15-item AEBS and scoring instructions are provided in the online Supplementary Materials.

### Internal consistency and descriptives (group 1)

Mean AEBS and subscale scores for group 1 are shown in Table 3. There were no differences between males and females on either subscale or on AEBS total scores (*P*_s_>0.182). Age did not correlate with scores on the appetitive drive subscale (*r*=−0.05, *P*=0.419), however small but significant negative correlations were observed between age and scores on the low-dietary control subscale (*r*=−0.22, *P<*0.001), and with the AEBS total score (*r*=−0.13, *P*=0.021). Cronbach’s alpha revealed high internal consistency for appetitive drive (*α*=0.90) and low dietary control scales (*α*=0.85).

### Internal consistency and descriptives (group 2)

Mean AEBS scores for group 2 are displayed in Table 3. AEBS total and subscale scores did not differ between groups 1 and 2 (*P*_s_>0.409). There were no gender differences on either subscale or on AEBS total scores in group 2 (*P*_s_>0.539). Age was negatively associated with scores on the appetitive drive subscale (*r*=−0.19, *P*=0.007), low dietary control subscale (*r*=−0.23, *P*=0.001) and total AEBS scores (*r*=−0.23, *P*=0.001). As in group 1, reliability estimates revealed high internal consistency for appetitive drive (*α*=0.85) and low-dietary control subscales (*α*=0.83).

### Confirmatory factor analysis (group 2)

**Figure 1:**
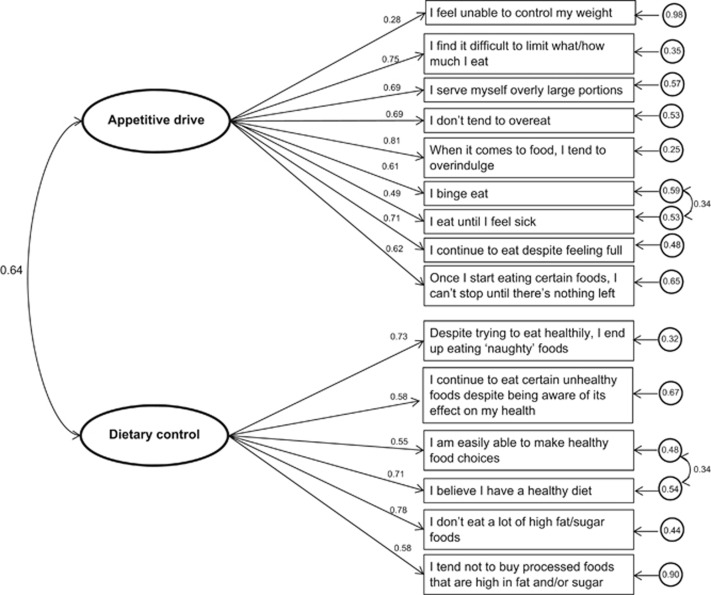
Factor model of AEBS with standardized factor loadings (i.e., values corresponding to one-way arrows), error terms (circled values) and covariances (values corresponding to two-way arrows).

Nine items were free to load onto the latent factor appetitive drive, and 6 items were free to load onto the latent factor low dietary control. The initial iteration indicated an acceptable to poor fit model (normed *χ*^2^ (*χ*^2^/df)=2.17, GFI=0.885, RMSEA (90% CI)=0.076 (0.061–0.091), CFI=0.910, SRMR=0.065). However, following the addition of covariance pathways based on modification indices (Figure 1), the two-factor model provided a good fit to the data (normed *χ*^2^ (*χ*^2^/df)=1.75, GFI=0.911, RMSEA (90% CI)=0.061 (0.044–0.077), CFI=0.944, SRMR=0.060). Standardized factor loadings indicated that all items appropriately reflected their underlying latent variable (*P*_s_<0.001) (Figure 1).

### Convergent and divergent validity (groups 1 and 2)

The AEBS total score correlated positively with all but the EAT-26 scale (Table 4), indicating good convergent validity. There was also evidence for overlap between the AEBS subscales and individual symptoms on the YFAS. In particular, scores on the low dietary control subscale were best predicted by the YFAS symptom ‘persistent desire or repeated unsuccessful attempts to quit’, whereas appetitive drive subscale scores were best predicted by the symptom ‘consume larger amounts than intended’ (see Online supplementary analysis for full results from this analysis). Furthermore, AEBS scores successfully predicted whether or not respondents perceived themselves to be food addicts, *B*=0.12, SE=0.01, odds ratio=1.13, *P<*0.001. Total AEBS scores did not correlate with scores on the BAS scale, indicative of good divergent validity. However small but significant correlations were observed between AEBS scores and the RAPI and BIS (Table 4).

### Incremental validity (groups 1 and 2)

After controlling for the variance accounted for by YFAS symptom count and BES scores, AEBS scores explained a significant proportion of additional variance in BMI (Table 5). AEBS and BES scores independently predicted BMI although the YFAS did not. Ordinal regression analyses revealed that the scale was able to predict the likelihood of being overweight and obese, independent of BES and YFAS scores (logit regression coefficient=0.03, s.e.=0.01, 95% CI=0.01, 0.06, Wald *χ*^2^=5.37, df=1, *P*=0.020, test of parallel lines: *P*=0.212). The odds ratio indicated that for every one unit increase in AEBS scores, the chances of an individual being classified as overweight or obese increased by 1.03. Notably, AEBS scores did not distinguish between underweight and normal weight participants (logit regression coefficient=0.00, 95% CI=−0.038, 0.038, Wald *χ*^2^=0.00, df=1, *P*=0.994). Weight classification was also significantly predicted by BES scores (logit regression coefficient=0.05, s.e.=0.02, 95% CI=0.02, 0.09, Wald *χ*^2^=8.10, df=1, *P*=0.004), but not by YFAS symptom count (logit regression coefficient=−0.12, s.e.=0.09, 95% CI=−0.30, 0.05, Wald *χ*^2^=1.97, df=1, *P*=0.160).

### Test–retest reliability (group 3)

Mean AEBS scores for group 3, at time 1 (t1) (that is, initial testing) and time 2 (t2) (that is, following a 2-week interval), are displayed in Table 3. The intra-class correlation coefficient revealed good test–retest reliability for each subscale (appetitive drive: *r*=0.74; low dietary control:*r*=0.74), and for AEBS total scores (*r*=0.77).

## Discussion

The current study developed and validated a novel tool, the AEBS, to assess the presence of behaviours which may underpin addiction-like patterns of eating. The AEBS comprised a two-factor scale structure, which was corroborated by a confirmatory factor analysis. Items in factor 1 referred to increased appetitive motivation, whereas items in factor 2 referred to low dietary control. Both subscales demonstrated good internal consistency, and good test–retest reliability over a 2-week interval. Mean scores on each subscale did not differ between males and females, however older age was associated with lower scores on the low dietary control subscale in both groups 1 and 2.

Notably, the two-factor structure of the AEBS is consistent with dual-process accounts of overeating and addictive behaviours.^44^ Specifically, enhanced reward responsivity is reflected by the ‘appetitive drive’ subscale, whereas the ‘low dietary control’ subscale reflects diminished top–down control. One possibility is that the enhanced appetitive drive in those with addiction-like eating may be partly due to diminished satiety signals and/or stronger perceptions of hunger. Indeed, several items in the AEBS reflect this (for example, ‘I find it difficult to limit what/how much I eat’ and ‘I serve myself overly large portions’), and previous research has demonstrated an attenuated decline in hunger following ingestion of a lunch meal in those with binge eating tendencies.^45^ However, the appetitive drive subscale also included items which explicitly refer to eating beyond physiological capacity (for example, ‘I continue to eat despite feeling full’) suggesting that it additionally captures behavioural and psychological features of overeating.

Indicative of good convergent validity, total AEBS scores correlated positively with other measures of maladaptive eating (that is, Emotional Eating Scale, Binge Eating Scale, YFAS symptom count) and BMI. The AEBS also significantly predicted whether or not individuals perceived themselves as ‘food addicts’. However, the scale failed to converge with a measure of disordered eating (that is, EAT-26). This is perhaps reflective of fundamental differences between the characteristics of traditional eating disorders (that is, anorexia nervosa, bulimia nervosa), and addiction-like eating patterns. Indeed, in our previous qualitative research,^18^ participants did not believe that food addiction was associated with weight and shape concern, periods of excessive food restriction or the tendency to engage in compensatory behaviours (for example, purging).

Crucially, the AEBS accounted for a significant proportion of variance in BMI above that predicted by the BES and YFAS. This is important as both of these measures assess patterns of eating that are thought to reflect ‘food addiction’.^6, 46^ Furthermore, the additional variance in BMI that was captured by the AEBS beyond the BES suggests that the scale successfully captures patterns of eating that are distinct from binge eating. In relation to this, previous research suggests that eating behaviour trait questionnaires tap into a common underlying factor (‘uncontrolled eating’) but at differing levels of severity.^47^ Specifically, measures of emotional eating and disinhibition captured intermediate degrees of uncontrolled eating, whereas the BES represented the most severe form. Applying this model to the current context, our results suggest that the AEBS may occupy a different part of the ‘uncontrolled eating’ continuum than the BES. Further research is needed to test this possibility and whether addiction-like eating patterns represent a more severe stage of uncontrolled eating than disinhibition and emotional eating.

Despite being significant independent predictors of BMI, AEBS and BES scores were highly correlated. It is therefore necessary to consider the extent to which manifestations of addiction-like eating, captured by the AEBS, are distinct from patterns of ‘binge’ eating. One imperative difference between binge eating and addiction-like eating behaviours may concern the timeframe in which overeating occurs. According to the DSM-5 criteria, binge eating disorder is characterised by a tendency to consume a large amount of food within a short space of time. In contrast, addiction-like eating may involve a more general tendency to overeat, or consume unhealthy foods, over longer time periods (for example, 4). Indeed, increased ‘grazing’ behaviour has been associated with eating pathology and poorer weight-loss outcomes following bariatric surgery (for example, refs 48, 49). In line with this, conceptualisations of food addiction, amongst members of the lay public, do not necessarily implicate the secretive and planned ‘binge’ episodes, and subsequent caloric restriction, that characterise binge eating disorder.^50, 51, 52^

An important distinction between the AEBS and previous measures of addictive eating (that is, YFAS and YFAS 2.0), is that the AEBS does not provide a dichotomous diagnostic criterion for eating addiction. As Ziauddeen *et al.*^5^ discuss, the limited consensus and understanding regarding exactly which behaviours (and their frequency/intensity) warrant a diagnosis of ‘eating addiction’, currently precludes the development of a diagnostic criterion. In addition, although psychometric tools offer the opportunity for screening and preliminary assessments, we agree with suggestions that the diagnosis of any psychological disorder should be reserved for trained clinicians, rather than self-report questionnaires.^53^ Further exploration of the characteristics of addiction-like eating behaviours is required to provide a diagnostic criterion that may be used within clinical settings.

The current study has several limitations. Firstly, while we attempted to recruit a representative community sample, respondents were predominantly female. Given that males and females may differ with regards to their conceptualisation of food addiction,^18^ further validation of the scale is required within a male population. Similarly, only 23% of the sample were overweight or obese (according to self-reports), and it is therefore possible that the characteristics of addiction-like eating identified in the AEBS may differ to those extant in overweight or clinical samples. Nonetheless, recent findings suggest that increased appetitive motivation and low self-control underpin a range of eating behaviour traits, but at differing levels of severity which correspond to increases in BMI.^17, 47^ Drawing upon these findings, we predict that obese samples would demonstrate similar patterns of addiction-like eating behaviour but at greater levels of severity. Future research is required to test this and to explore the scale’s ability to predict BMI in those with obesity.

A second limitation is that the current study used a cross-sectional design, and thus we were unable to draw conclusions about the causal relationship between AEBS scores and BMI. Therefore, the extent to which the scale is predictive of prospective weight gain and weight-loss success are important avenues for future research. It would also be interesting to examine whether addiction-like eating may arise following attempts at dietary control and food restriction. However, we suggest that increased reward responsivity to food following dietary restriction represents an adaptive mechanism, and so we would not expect the AEBS to capture such behaviours. In support of this, the scale did not distinguish between underweight (who likely consume fewer calories than their metabolic requirements) and normal weight participants, nor did it correlate with scores on the EAT-26 (which includes items relating to dietary restriction). These findings suggest that the AEBS captures maladaptive patterns of eating that predispose people to having a higher BMI.

It is also important to note that measures of height and weight were obtained via self-report. This may have limited the accuracy of the BMI data as individuals tend to overestimate their height and underestimate their weight.^54^ Despite this, self-reported height and weight have been found to correlate strongly with measurements obtained by a researcher and thus are thought to provide valid estimates of anthropometric data.^54^

Finally, scale items were derived primarily from public perceptions of food addiction which may not accurately reflect scientific understanding of the processes involved in addictive behaviours. However, contrary to this concern, the two-factor scale structure that emerged reflects well-established dual-process models of overeating and addiction,^17^ suggesting that items included in the AEBS are consistent with theoretical models of motivated behaviours.

In conclusion, the AEBS represents a valid and reliable tool to assess addiction-like eating behaviours in community samples. By focusing on core behavioural features of a potential ‘eating addiction’, the AEBS overcomes many of the limitations associated with applying the diagnostic criteria for substance dependence to eating behaviour. Critically, the AEBS was able to successfully predict a significant proportion of variance in BMI above that predicted by the YFAS and BES. Future research is required to validate the AEBS within obese and weight-management populations, and establish clinically meaningful cut-off points for the scale. In doing so, the AEBS has important implications for the identification, prevention, and treatment of those at risk of overeating and obesity.

## Figures and Tables

**Table 1:** Characteristics of participants in each group

	*Group 1 (*n=*307)*	*Group 2 (*n=*204)*	*Group 3 (*n=*70)*
Females/males	270/37	170/34	39/31
Age (years): mean (s.d.)	24.32 (±10.69)	24.03 (±11.18)	36.63 (±15.14)
Age (years): range	18–67	18–66	18–86
BMI (kg m^−2^): mean (s.d.)	23.58 (±5.12)	23.24 (±5.07)	25.81 (±4.57)
BMI (kg m^−2^): range	15.41–53.12	15.20–60.26	15.75–36.67
Overweight/obese (*n*)	45/30	29/16	29/12

Values in parentheses represent the standard deviation (±s.d.) of the mean.

**Table 2:** Factors, items and factor loadings

*Factor*^a^	*Item (response format)*	*Factor loadings*
Appetitive drive	I continue to eat despite feeling full (never-always)	0.826
	I serve myself overly large portions (never-always)	0.818
	I find it difficult to limit what/how much I eat (never-always)	0.796
	Once I start eating certain foods, I can’t stop until there’s nothing left (never-always)	0.783
	When it comes to food, I tend to overindulge (never-always)	0.733
	I don't tend to overeat^b^ (strongly disagree-strongly agree)	0.702
	I feel unable to control my weight (strongly disagree-strongly agree)	0.618
	I binge eat (never-always)	0.639
	I eat until I feel sick (never-always)	0.606
Low dietary control	I tend not to buy processed foods that are high in fat and/or sugar^b^ (strongly disagree-strongly agree)	0.818
	I don't eat a lot of high fat/sugar foods^b^ (strongly disagree-strongly agree)	0.823
	I believe I have a healthy diet^b^ (strongly disagree-strongly agree)	0.798
	I am easily able to make healthy food choices^b^ (never-always)	0.736
	Despite trying to eat healthily, I end up eating ‘naughty’ foods (never-always)	0.640
	Despite being aware of its effect on my health (never-always), I continue to eat certain unhealthy foods	0.610

a Critically, factors were not determined by the different response formats used (that is, ‘never-always’/‘strongly disagree-strongly agree’).

b Items were reverse scored prior to analyses.

**Table 3:** AEBS total and subscale scores for each of the three groups

	*Group 1 (*n=*307)*	*Group 2 (*n=*204)*	*Group 3 (t1)*^a^ (n=*70)*	*Group 3 (t2)*^a^
AEBS total^b^	41.41 (±9.83)	40.95 (±9.05)	41.39 (±9.95)	40.91(±10.03)
AEBS (appetitive drive)^c^	23.51 (±6.73)	23.05 (±5.88)	23.61 (±5.91	23.10 (±6.21)
AEBS (low dietary control)^d^	17.90 (±4.46)	17.90 (±4.37)	17.77 (±4.54)	17.81 (±4.41)

a t1 refers to scores obtained at the initial time of testing; t2 refers to scores obtained following a 2-week interval.

b AEBS total scores range from 15 (minimum) to 75 (maximum).

c AEBS appetitive drive scores range from 9 (minimum) to 45 (maximum)

d AEBS low dietary control scores range from 6 (minimum) to 30 (maximum).

Values are means±s.d.s.

**Table 4:** Descriptive statistics and correlations with AEBS (*N*=511)

*Variable*	M(±*s.d.)*	*Cronbach’s*α	*Correlation (*r*) with AEBS*	P
Binge eating scale	10.81 (±8.00)	0.91	0.67	<0.001
YFAS (symptoms)^a^	2.08 (±1.51)	0.90	0.56	<0.001
EES	52.93 (±18.03)	0.94	0.47	<0.001
EAT-26	8.30 (±7.99)	0.89	0.05	0.288
BMI (kg m^−2^)	23.45 (±5.10)		0.26	<0.001
RAPI	7.60 (±9.47)	0.92	0.22	<0.001
BIS	19.23 (±2.30)	0.79	0.15	<0.001
BAS	37.62 (±5.07)	0.85	0.05	0.293

Abbreviations: BAS Behavioural Activation Scalev; BIS Behavioural Inhibition Scale; EAT-26 Eating Troubles Module; EES Emotional Eating Scale; RAPI Rutgers Alcohol Problem Index; YFAS Yale Food Addiction Scale.

a 46 (9%) participants from groups 1 and 2 fulfilled the YFAS criteria for food addiction.

**Table 5:** Hierarchical multiple regression showing the YFAS and BES symptom count (step 1) and AEBS (step 2) as predictors of BMI

	*Cumulative*		*Simultaneous*			
	*F-change*	R^2^*-change*	β	SR^2^	P	*95% Confidence interval*
Step 1	F_(2, 500)_=23.44**	0.09				
YFAS (symptoms)			−0.07	−0.11	0.208	−0.64–0.14
BES			0.34**	0.06	<0.001	0.14–0.29
Step 2	F_(1, 499)_=4.93*	0.01				
AEBS			0.13*	0.01	0.027	0.01–0.13

*Note.* SR^2^ is the squared semi-partial correlation. *P<0.05, **P<0.001. Variance accounted for by the full regression model: *R*^2^=0.10, F_(3, 502)_=17.39, *P*<0.001.

N.B.: All tolerance and VIF values were within the commonly accepted cut-off criteria (that is, tolerance >0.20; VIF<4.0), indicating no problems with multi-collinearity.^55^
