# Supplementary material for: The development and validation of the Addiction-like Eating Behaviour Scale
Source: Int J Obes (Lond). 2017 Aug 8;41(11):1710–7. doi: 10.1038/ijo.2017.158 (PMC5682562; doi:10.1038/ijo.2017.158)
Supplement: Supplementary Information [file ijo2017158x1.docx]

# Supplementary Online Materials: The Development and Validation of the Addiction-like Eating Behaviour Scale

Contents

[1. Assessment of convergent validity 2](#_Toc485042741)

[*1.1.* *Yale Food Addiction Scale* 2](#_Toc485042742)

[*1.2.* *Binge Eating Scale* 2](#_Toc485042743)

[*1.3.* *Emotional Eating Scale* 3](#_Toc485042744)

[*1.4.* *Eating Troubles Module* 3](#_Toc485042745)

[*1.5.* *Assessment of self-perceived ‘food-addiction’.* 3](#_Toc485042746)

[2. Assessments of divergent validity 4](#_Toc485042747)

[*2.1.* *Rutgers Alcohol Problem Index* 4](#_Toc485042748)

[*2.2.* *Behavioural Inhibition System/Behavioural Approach System Reactivity* 4](#_Toc485042749)

[3. Divergent validity: Results and discussion 4](#_Toc485042750)

[4. Comparison with YFAS 4](#_Toc485042751)

[5. The Addiction-like Eating Behaviour Scale (AEBS) 7](#_Toc485042752)

[6. AEBS scoring instructions 8](#_Toc485042753)

[References 8](#_Toc485042754)

## Assessment of convergent validity

The following measures were used to assess the convergent validity of the AEBS:

- 1. *Yale Food Addiction Scale* (YFAS; 1). The YFAS consists of 25 items designed to measure an addiction to foods high in fat and/or sugar. The scale is based on the DSM-IV criteria for substance dependence. For the first 16 items, a Likert scale is used in which the respondent indicates how often, in the past 12 months, they have engaged in a particular behaviour (for example “I eat to the point where I feel physically ill”). For the next 9 items, respondents indicate whether or not they agree with each statement by marking either ‘Yes’ or ‘No’ (for example, “I want to cut down or stop eating certain kinds of foods”). Again, respondents are asked to base their response on their experiences in past 12 months. Respondents are also asked to indicate all foods that they have ‘problems’ with. A diagnosis of food addiction is given when the individual demonstrates significant clinical impairment due to the their eating behaviours, and fulfils at least 3 of the following symptoms: unsuccessful attempts to quit, giving up activities to eat, eating large portions, continuing to overeat despite negative consequences, tolerance to food, withdrawal from not eating, and spending a lot of time eating. The YFAS also provides a continuous measure of the number of food addiction symptoms exhibited by an individual (i.e. symptom count) which range from 0 to 7.
  2. *Binge Eating Scale* (BES; 2). The BES consists of 16 items which assess the severity of binge eating symptoms. Each item consists of three or four statements about eating behaviours or emotions associated with binge-eating. Instructions are given to mark the statement within each item which the participant most identifies with. Higher scores on the BES indicate more severe binge eating symptoms. This scale was selected on the basis that it was used to assess the convergent validity of a previous measure of food addiction (i.e. the Yale Food Addiction Scale, 1).
  3. *Emotional Eating Scale* (EES; 3). The EES consists of 25-items which assess tendencies to engage in emotionally-driven overeating. A list of 25 mood states is provided (e.g. lonely, bored), and respondents are required to indicate the extent to which each mood would initiate overeating (ranging from ‘no desire’ to ‘an overwhelming desire’). This measure has previously been used to assess the convergent validity of the YFAS (1).
  4. *Eating Troubles Module* (EAT-26; 4). The EAT-26 is based on the Eating Attitudes Test and provides a measure of the signs and symptoms associated with eating disorders. A high risk of disordered eating is indicated by scores equal to or greater than 20. This measure was selected on the basis that it has previously been used to assess the convergent validity of the YFAS, (1).
  5. *Assessment of self-perceived ‘food-addiction’.* As in Ruddock et al. (5), to assess self-perceived food addiction, participants were asked ‘Do you agree with the following statement: “I believe myself to be a food addict”?’. Participants were required to tick either ‘yes’ or ‘no’. Previously, we found that individuals who perceived themselves to be addicted to food demonstrated increased food reward, and consumed more calories from a high-fat food, compared to those who did not perceive themselves as ‘food addicts’ (5).

## Assessments of divergent validity

The following measures were included to assess the scale’s divergent validity, and thus were *not* expected to correlate with AEBS scores:

- 1. *Rutgers Alcohol Problem Index* (RAPI; 6). The RAPI consists of 23-items which assess drinking problems within young adults. This measure was selected on the basis that is has previously been used to assess the divergent validity of the YFAS (1).
  2. *Behavioural Inhibition System/Behavioural Approach System Reactivity* (BIS/BAS; 7). The BIS/BAS questionnaire consists of 20-items which assess the Behavioural Inhibition (BIS) and Behavioural Approach Systems proposed by Gray (8). The instrument provides a total BIS score and three BAS-related subscale scores: Drive (DRV), Fun Seeking (FS), and Reward Responsiveness (RR). This measure has previously been used to assess the divergent validity of the YFAS (1).

## Divergent validity: Results and discussion

The AEBS was unrelated to scores on the BAS, indicative of good divergent validity. However the AEBS correlated positively with the BIS and the RAPI. Similar positive associations between the BIS and eating behaviour have been reported previously (9), and perhaps reflect a tendency to eat in response to negative affect. Furthermore, the positive correlation between the AEBS and RAPI may be explained by common personality attributes (e.g. impulsivity) that have been associated with alcoholism, obesity, and eating disorders (10,11).

## Comparison with YFAS

Table S1 shows YFAS symptom frequency across groups 1 and 2. These rates are similar to those obtained in previous research (e.g. 12). The strongest predictor of AEBS appetitive drive subscale scores was the tendency to ‘Consume larger amounts than intended’. Scores on the low dietary control subscale were best predicted by the YFAS symptom ‘Persistent desire or repeated unsuccessful attempts to quit’. Nine percent (N=46) of the total sample met the YFAS criteria for food addiction. YFAS diagnosed food addiction significantly predicted scores on both subscales (appetitive drive subscale: *ß*=.27, 95% confidence intervals (CIs): 4.17 - 7.92, *p*<.001; Low dietary control: *ß*=.14, 95% confidence intervals (CIs): .77 – 3.42, *p*=.002).

Table S1. *Linear regression showing YFAS symptoms as predictors of each AEBS subscale. The number (n) and percentage of the sample meeting the criteria for each YFAS symptom is also shown.*

|  |  | Regression for low dietary control subscale | | | | Regression for appetitive drive subscale | | | |
| --- | --- | --- | --- | --- | --- | --- | --- | --- | --- |
| Symptom | *n*(%) | ß | *p* | SR^2^ | 95% confidence intervals | ß | *p* | SR^2^ | 95% confidence intervals |
| Consume larger amounts than intended | 40 (8%) | .13* | .003 | .02 | .73 – 3.60 | .28** | <.001 | .09 | 4.74 - 8.39 |
| Persistent desire or repeated unsuccessful attempts to quit | 463(91%) | .21** | <.001 | .05 | 2.02 – 4.46 | .15** | <.001 | .03 | 1.78 – 4.89 |
| Much time/activity to obtain, use, recover | 115(23%) | .13* | .003 | .02 | .49 – 2.34 | .19** | <.001 | .04 | 1.74 - 4.09 |
| Important social, occupational, or recreational activities given up or reduced | 88(17%) | .12* | .017 | .01 | .24 – 2.50 | .11* | .012 | .01 | .41 - 3.29 |
| Use despite knowledge of adverse consequences | 142(28%) | .07 | .115 | .01 | -.17 – 1.58 | .13* | .002 | .02 | .66 - 2.90 |
| Tolerance (marked increase in amount; marked decrease in effect) | 128(25%) | .06 | .164 | .00 | -.26 – 1.54 | .10* | .011 | .01 | .35 - 2.65 |
| Characteristic withdrawal symptoms; substance taken to relieve withdrawal symptoms | 85(17%) | .06 | .202 | .00 | -.40 – 1.90 | .11* | .009 | .01 | .50 - 3.43 |
| Use causes clinically significant impairment or distress | 57(11%) | -.08 | .125 | .00 | -2.44 - .30 | -.03 | .472 | .00 | -2.39 – 1.11 |

*Note.* SR^2^ is the squared semi-partial correlation. *p<.05 **p<.001. Variance accounted for by the full regression model: *R^2^=.*37, *F*(8,502)=36.02, *p*<.001

## The Addiction-like Eating Behaviour Scale (AEBS)

|  | Never | Rarely | Sometimes | Most of the time | Always |
| --- | --- | --- | --- | --- | --- |
| 1. I continue to eat despite feeling full |  |  |  |  |  |
| 2. I serve myself overly large portions |  |  |  |  |  |
| 3. I find it difficult to limit what/how much I eat |  |  |  |  |  |
| 4. I binge eat |  |  |  |  |  |
| 5. When it comes to food, I tend to over-indulge |  |  |  |  |  |
| 6. I am easily able to make healthy food choices |  |  |  |  |  |
| 7. Once I start eating certain foods, I can’t stop until there’s nothing left |  |  |  |  |  |
| 8. Despite trying to eat healthy, I end up eating ‘naughty’ foods |  |  |  |  |  |
| 9. I eat until I feel sick |  |  |  |  |  |
| 10. I continue to eat certain unhealthy foods despite being aware of their effects on my health |  |  |  |  |  |
|  | **Strongly disagree** | **Disagree** | **Neither agree/disagree** | **Agree** | **Strongly agree** |
| 11. I tend NOT to buy processed foods that are high in fat, salt, & sugar |  |  |  |  |  |
| 12. I don’t eat a lot of high fat/sugar foods |  |  |  |  |  |
| 13. I believe I have a healthy diet |  |  |  |  |  |
| 14. I don’t tend to overeat |  |  |  |  |  |
| 15. I feel unable to control my weight |  |  |  |  |  |

## AEBS scoring instructions

Each item is given a score ranging from 1 (‘Never’ or ‘Strongly disagree’) to 5 (‘Always’ to ‘Strongly Agree’). Items 6, 11, 12, 13, 14 are REVERSE scored (i.e. ‘Always/Strongly agree’ = 1; and ‘Never/Strongly disagree’ =5).

The scale provides total score (max score = 75), and 2 subscale scores:

Appetitive drive subscale (max score = 45): Items 1-5, 7, 9, 14, and 15.

Low dietary control subscale (max score = 30): Items 6, 8, 10, 11, 12, 13.

### References

1. Gearhardt AN, Corbin WR, Brownell KD. Preliminary validation of the Yale Food Addiction Scale. *Appetite* 2009*;* **52**(2); 430–436.
2. Gormally J, Black S, Daston S, Rardin D. The assessment of binge eating severity among obese persons. *Addict Behav* 1982; **7**(1): 47–55.
3. Arnow B, Kenardy J, Agras WS. The Emotional Eating Scale: The Development of a Measure to Assess Coping with Negative Affect by Eating. *Int J Eat Disord* 1995; **18**(1): 79–90.
4. Garner DM, Olmsted MP, Bohr Y, Garfinkel PE. The eating attitudes test: psychometric features and clinical correlates. *Psychol Med* 1982; **12**: 871–878.
5. Ruddock HK, Field M, Hardman CA. Exploring food reward and calorie intake in self-perceived food addicts. *Appetite* 2016 (in press). DOI: [10.1016/j.appet.2016.12.003](http://doi.org/10.1016/j.appet.2016.12.003)
6. White HR, Labouvie EW. Toward the assessment of adolescent problem drinking. *J Stud Alcohol* 1989; **50**: 30–37.
7. Carver CS, White TL. Behavioral Inhibition, Behavioral Activation, and Affective Responses to Impending Reward and Punishment: The BIS/BAS Scales. *J Pers Soc Psychol* 1994; **67**(2): 319-333.
8. Gray JA. The psychology of fear and stress. Cambridge: Cambridge University Press; 1987.
9. Dietrich A, Federbusch M, Grellmann C, Villringer A, Horstmann A. Body weight, eating behavior, sensitivity to reward/punishment, and gender: relationships and interdependencies. *Front Psychol* 2014; **5**(1073): 1-13.
10. Fischer S, Smith GT. Binge eating, problem drinking, and pathological gambling: Linking behavior to shared traits and social learning. *Pers Individ Dif* 2008; **44**(4): 789-800.
11. Fischer S, Smith GT, Anderson KG. Clarifying the role of impulsivity in bulimia nervosa. *Int J Eat Disord* 2003; **33**(4): 406–411.
12. Schulte EM, Avena NM, Gearhardt AN. Which foods may be addictive? The roles of processing, fat content, and glycemic load. PLoS One. 2015 Feb 18;10(2):e0117959.
